# MAKING MOVES: EPIGENETIC AGING AND ITS RELATIONSHIP WITH PAIN AND DISABILITY OUTCOMES IN OLDER ADULTS

**DOI:** 10.1093/geroni/igae098.0438

**Published:** 2024-12-31

**Authors:** Larissa Strath, Ania Lipat, Lingsong Meng, Zhiguang Huo, Thomas Foster, Alisa Johnson, Roger Fillingim, Yenisel Cruz-Almeida

**Affiliations:** University of Florida, Gainesville, Florida, United States; University of Florida, Gainesville, Florida, United States; University of Florida, Gainesville, Florida, United States; University of Florida, Gainesville, Florida, United States; University of Florida, Gainesville, Florida, United States; University of Florida, Gainesville, Florida, United States; University of Florida, Gainesville, Florida, United States; University of Florida, Gainesville, Florida, United States

## Abstract

Chronic pain (pain that is persistent or recurrent for 3 months or longer) is a leading cause of mobility interference and disability in the aging population. It is well known that chronological aging increases the risk of developing of pain and pain reduces physical function; however, limited data exist on how epigenetic aging, new hallmark of biological aging shown to predict pain and mobility outcomes. The purpose of this session is three-fold: 1) introduce the audience to the algorithmic epigenetic aging clocks; 2) examine whether decreased mobility and increased pain severity is associated with these epigenetic clocks, and 3) examine recent literature showing epigenetic age acceleration as a potential mechanism by which biopsychosocial factors could be influencing pain and mobility in older adults. Given the complex multi-dimensional experience of pain and disability that is dynamically sculpted throughout an individual’s lifespan along with the environmental interactions with the epigenome, these advances will be a welcomed approach and the future of personalized medicine in an aging population.

